# Gratitude at Work: Prospective Cohort Study of a Web-Based, Single-Exposure Well-Being Intervention for Health Care Workers

**DOI:** 10.2196/15562

**Published:** 2020-05-14

**Authors:** Kathryn C Adair, Larissa G Rodriguez-Homs, Sabran Masoud, Paul J Mosca, J Bryan Sexton

**Affiliations:** 1 Duke Center for Healthcare Safety and Quality Duke University Health System Durham, NC United States; 2 Duke University School of Medicine Durham, NC United States; 3 Duke Network Services Duke University Health System Duke University Health System Durham, NC United States; 4 Department of Surgery Duke University School of Medicine Duke University Health System Durham, NC United States; 5 Department of Psychiatry Duke University School of Medicine Duke University Health System Durham, NC United States

**Keywords:** burnout, health care, positive psychology, mental health, emotions

## Abstract

**Background:**

Emotional exhaustion (EE) in health care workers is common and consequentially linked to lower quality of care. Effective interventions to address EE are urgently needed.

**Objective:**

This randomized single-exposure trial examined the efficacy of a gratitude letter–writing intervention for improving health care workers’ well-being.

**Methods:**

A total of 1575 health care workers were randomly assigned to one of two gratitude letter–writing prompts (self- vs other focused) to assess differential efficacy. Assessments of EE, subjective happiness, work-life balance, and tool engagement were collected at baseline and 1-week post intervention. Participants received their EE score at baseline and quartile benchmarking scores. Paired-samples *t* tests, independent *t* tests, and correlations explored the efficacy of the intervention. Linguistic Inquiry and Word Count software assessed the linguistic content of the gratitude letters and associations with well-being.

**Results:**

Participants in both conditions showed significant improvements in EE, happiness, and work-life balance between the intervention and 1-week follow-up (*P*<.001). The self-focused (vs other) instruction conditions did not differentially predict improvement in any of the measures (*P*=.91). Tool engagement was high, and participants reporting higher motivation to improve their EE had higher EE at baseline (*P*<.001) and were more likely to improve EE a week later (*P*=.03). Linguistic analyses revealed that participants high on EE at baseline used more negative emotion words in their letters (*P*=.005). Reduction in EE at the 1-week follow-up was predicted at the level of a trend by using fewer first-person (*P*=.06) and positive emotion words (*P*=.09). No baseline differences were found between those who completed the follow-up assessment and those who did not (*P*s>.05).

**Conclusions:**

This single-exposure gratitude letter–writing intervention appears to be a promising low-cost, brief, and meaningful tool to improve the well-being of health care workers.

## Introduction

### Background

Emotional exhaustion (EE) among US health care workers has increased to 1 out of 3 nurses [1] and almost half of practicing physicians [2], with the highest rates among junior doctors and those working at the front line of patient care [3,4]. Many aspects of patient safety and quality of care appear compromised by EE, which is a key component of burnout [5]. EE has been linked to higher frequencies of medical errors, lapses in professionalism, impeded learning, and suicidal ideation [6-8]. As such, there is an urgent need for simple, brief, effective, and accessible EE and burnout interventions on a broad scale.

Unfortunately, existing remedies for EE are limited by time, effort, and cost. For instance, mindfulness-based meditation courses have empirical support for reducing EE [9]; however, courses of this type can be costly and typically require 8 to 10 weeks and approximately 90 hours of effort from health care workers who already skip meals and breaks, deprive themselves of needed rest, and get home late as a norm [10,11]. In contrast, this study examines the efficacy of a brief, single-use tool—writing a letter of gratitude—for reductions in EE and improvements in well-being. If there is a way to use bite-sized tools to provide a quick recharge in the face of EE, it could facilitate (not replace) the use of other, more elaborate remedies.

Gratitude has one of the strongest associations with better mental health and well-being of any personality trait, even more than hope, optimism, or compassion [12]. Several rigorous controlled trials demonstrate the beneficial effect of gratitude interventions [13-15]. For instance, participants randomized to gratitude journal keeping, compared with active control conditions, reported higher levels of energy, determination, enthusiasm, feeling more optimistic, being more likely to exercise, having fewer physical symptoms, sleeping longer, and sleeping with better quality [16]. Another study found that participants randomized to write a gratitude letter and visit the recipient of the letter reported significant gains in happiness and reductions in depressive symptoms, compared with a placebo control group, both 1 week and 1 month later [17]. In fact, this intervention exhibited the greatest benefits compared with four other positive psychology interventions that the authors tested (ie, 3 good things, you at your best, using signature strengths, and identifying signature strengths) [17].

Previous research on live interactions has shown that expressions of gratitude that focused more on the benefits that the person expressing the gratitude received (ie, self-focused) benefited the expresser, whereas gratitude focusing more on the positive attributes of the individual being thanked (ie, other focused) benefited the recipient of said gratitude [18]. We hypothesized that participants randomized to gratitude letter–writing instructions to focus more on personal benefits in their letters (ie, self-focus) would exhibit greater improvement in well-being than those randomized to focus more on the positive aspects of the letter recipient (ie, other-focused).

On the basis of previous linguistic studies that have identified a *language of depression*, we were interested in identifying a *language of burnout* within the gratitude letters [19]. As such, we developed a series of hypotheses regarding the frequencies of words in particular word categories and their associations with both concerning levels of baseline EE and EE improvement. More frequent use of first-person singular (eg, *I*) and negative emotion words and marginally fewer positive emotion words have been identified in those experiencing depression [19,20]. Greater use of cognitive processing words (eg, *because* and *realization*) predicts fewer posttraumatic stress disorder symptoms among trauma survivors [21], and in our own research, third-person plural use (eg, *we*) is higher in groups with lower EE [22]. Taking this research together, we expected participants with concerning levels of EE at baseline to use more first-person singular and negative emotion words and fewer third-person plural, positive emotion, and cognitive processing words in their letters. We expected that EE improvers would exhibit the inverse of this pattern.

### Objectives

This trial examined the efficacy of a gratitude letter–writing intervention to improve health care workers’ well-being measured in terms of EE, happiness, and work-life balance. The aims of this study were (1) to determine whether a onetime gratitude letter predicts improvements in well-being and to test whether self- vs other-focused instruction conditions differentially predict changes in well-being, (2) to measure participants’ engagement with the intervention and their reactions to receiving feedback on their individual EE, and (3) to examine the linguistic content of gratitude letters for correlates of baseline EE and predictors of EE improvement.

## Methods

### Design and Patient Population

This randomized before-and-after trial of a gratitude letter–writing intervention was conducted between January 2018 and February 2019 (institutional review board approval: Pro00063703). Health care workers enrolled through a website link [23], having learned of the study through colleagues or by participating in a workshop conducted by authors KA and JS. All health care workers (clinical and nonclinical) aged at least 18 years were eligible to participate. Participants completed assessments and the gratitude letter–writing intervention at baseline and were given the opportunity to complete assessments again 1 week later. The intervention and assessments were completed on the web.

Among the 1575 health care workers who participated in the gratitude letter–writing intervention, 1179 (74.86%) wrote 15 words or more, a threshold at which we found letters were less likely to be left unfinished*.* A total of 17.59% (227/1575) participants completed both the pre- and postintervention assessments.

### Measures

A total of 3 validated scales were used to assess aspects of well-being: EE, subjective happiness, and work-life balance. These scales were selected for the following reasons: (1) all are brief (8 items or less) and therefore are less onerous to busy health care workers to complete, (2) all are psychometrically sound both in prior studies and in our own, and (3) all are sensitive to intervention in our prior studies [24]. Demographic questions on gender, race, ethnicity, and role were included at baseline.

#### Emotional Exhaustion

The Maslach Burnout Inventory (MBI) is widely used among professionals in human services, including health care workers, and is considered the gold standard survey instrument for assessing professional burnout [25]. The MBI includes 3 scales: EE, depersonalization, and personal accomplishment. EE consistently produces the largest and most consistent coefficient alpha estimates compared with depersonalization and personal accomplishment [5,26,27]. EE has also been used to discriminate between burned-out and nonburned-out outpatients suffering from work-related neurasthenia (according to International Classification of Diseases-10 criteria and Diagnostic and Statistical Manual of Mental Disorders-IV) [27,28]. We used a 5-item derivative of the original 9-item EE scale [25,29]. An example item is “I feel frustrated by my job.” Participants respond using a 5-point scale (1=disagree strongly and 5=agree strongly), and mean scores are rescaled to 0 to 100, with higher scores signifying more EE. Internal consistency in this study was good (Cronbach alpha at baseline=.84 and Cronbach alpha at follow-up=.86).

To examine linguistic differences by level of EE, we compared the language of a priori selected word categories for those participants whose EE scores reflected moderate and severe EE (*concerning*) with participants without EE. An EE score greater than or equal to 75 was deemed concerning, which reflects, on average, agreeing slightly or strongly to EE items. The concerning threshold should not be considered clinically diagnostic, but rather, it identifies those whose scores suggest a pronounced level of EE [29]. Those determined as not having EE (not concerning) had scores less than 50, which reflects, on average, disagreeing slightly or strongly to EE items. Participants who scored between 50 and 75 (mild EE) were not included in these analyses. To compare linguistic markers of EE improvement, we identified *improvers* as having a reduction of their burnout score of more than 10 points and *decliners* as having scores that increased by 10 or more points.

#### Subjective Happiness

Lyubomirsky and Lepper’s Subjective Happiness Scale (SHS) is a 4-item internationally used scale of global happiness [30,31]. An example item is “In general I consider myself (1=not a very happy person to 7=a very happy person)”. SHS items are answered using a 7-point scale, and each participant’s responses are averaged, with higher scores indicating higher happiness. Internal consistency in this study was good (Cronbach alpha at baseline=.83 and Cronbach alpha at follow-up=.87).

#### Work-Life Balance

The work-life climate scale evaluates individual differences in work-life infraction behaviors and has been shown to have good psychometrics when administered to health care workers [10,11,29,32]. The scale prompt asks *During the past week, how often did this occur*? Items include: *Arrived home late from work; Slept less than 5 hours in a night.* Responses include rarely or none of the time (less than 1 day), some or a little of the time (1-2 days), occasionally or a moderate amount of time (3-4 days), all of the time (5-7 days), and not applicable. Scale scores were computed by taking the mean of the items [10]. Internal consistency in this study was acceptable (Cronbach alpha at baseline=.71 and Cronbach alpha at follow-up=.77).

#### Intervention Experience

In addition, five questions assessed participants’ experience with the tool. In the baseline survey, these questions were “Would you like a copy of this letter emailed to you?” (yes, no, or do not know) and “I completed this gratitude letter exercise before” (yes or no). The 1-week follow-up surveys included “Do you have other people in mind for whom you might write a letter like this?” (yes, no, or not sure), “Did you talk to anyone about your first gratitude letter”? (yes, no, or not sure), and “Since my first gratitude letter a week ago, it has gotten easier to think of things for which I am grateful” (1=very strongly agree and 8=very strongly disagree).

### Emotional Exhaustion Feedback

After completing the items for the EE scale, participants were shown their scores and the quartiles of scores from our sample of over 135,000 US health care workers. Participants answered three Likert-like questions after receiving their scores and before the gratitude letter–writing intervention: “I was surprised by my burnout score,” “Knowing my burnout score makes me want to work on it more,” and “I would like to be more resilient.” Response options ranged from 1 to 5 (disagree strongly to agree strongly).

### Gratitude Intervention

The gratitude letter–writing intervention invited participants to spend approximately 7 min writing an appreciative letter to someone who has positively affected their lives.

Participants were given general instructions plus two text boxes (parts 1 and 2) to enter their letters. Participants were randomized, through an automatic randomizer within the survey software program, to receive either the *self-focus* condition or the *other-focus* condition as described below:

Think of someone who has done something amazing for you; this person can be alive or no longer with us. This person contributed to your well-being in a big way. Spend the next 7 minutes writing a genuine, kind and appreciative 2-part note:

Self-focus condition:

Part 1:Tell this person what they did, how it impacted you, and the benefits you received.

Part 2: Tell this person why it was important to you.

Other-focus condition:

Part 1: Tell this person what they did, how it impacted you, how it made you feel, and why it was important to you.

Part 2: Tell this person what it says about them, that they did this amazing thing for you. You might include what this says about your relationship to this person.

### Statistical Analysis

Demographic variables and evaluation questions were subjected to descriptive analysis. Cronbach alpha assessed the internal reliability of administered scales, with values above .70 considered acceptable [33]. Paired *t* tests assessed improvements in well-being between baseline and 1-week postintervention surveys. Independent-samples *t* tests compared changes in well-being scores between baseline and 1-week follow-up between gratitude letter–writing conditions. A manipulation check compared the linguistic use of first-person singular (eg, *I*) vs second person (eg, *you*) based on condition; the self-focus condition was expected to use more first-person words and fewer second-person words than the other-focus condition. Independent-samples *t* tests also compared EE feedback scores based on EE level at baseline (concerning vs no EE), EE improvement (improvers vs decliners), and baseline well-being scores in those who completed 1-week postintervention surveys vs those who did not. *P* values less than .05 were considered significant, and all tests were 2 tailed. Listwise deletion was used for missing data. Analyses were performed using SPSS version 24 software.

### Linguistic Analysis of Letters

The Linguistic Inquiry and Word Count (LIWC) software program version 2015 was used to analyze text written in the participants’ gratitude letters [34]. The LIWC dictionary consists of 6400 words, word stems, and select emotions, with each entry defining one or more of 80 language categories [35]. Word counts are expressed as a percentage of the total number of words, controlling for the length of the text file. Text from both parts 1 and 2 of the instructions were included in the linguistic analyses.

Independent-samples *t* tests compared differences in linguistic categories based on EE level at baseline (concerning vs no EE) and also compared differences in linguistic word count frequencies for EE improvers and decliners.

## Results

### Respondent Demographics

Overall, 1575 participants completed the onetime gratitude intervention and baseline assessments. The majority of participants were white and female (1165/1575, 73.84% and 1100/1575, 69.84%, respectively). The top 3 health care worker roles were those who classified as *other* (334/1575, 21.21%), *attending or staff physician* (266/1575, 16.89%), and *other manager* (260/1575, 16.51%). Nurse managers and charge nurses, when combined, accounted for 21.71% (342/1575). See Table 1 for additional demographic information.

Overall, 277 participants (self-focused condition=139 and other-focused condition=138) provided correct contact information and followed up at 1 week using a onetime email prompt. An independent *t* test revealed no differences in baseline EE, subjective happiness, or work-life balance for those who completed the 1-week follow-up and those who did not (*P*=.48, *P*=.44, and *P*=.12, respectively). At baseline, 38.02% (579/1523) participants met or exceeded the threshold for having concerning levels of EE.

**Table 1:** Respondent demographics of gratitude letter–writing participants (N=1575).

Demographics		Participants, n (%)
**Health care worker role**		
	Other	334 (21.21)
	Attending or staff physician	266 (16.89)
	Fellow physician	6 (0.38)
	Resident physician	6 (0.38)
	Physician assistant, nurse practitioner, clinical nurse specialist	58 (3.68)
	Nurse manager or charge nurse	175 (11.11)
	Registered nurse (including certified registered nurse anesthetists)	167 (10.60)
	Pharmacist	138 (8.76)
	Therapist (respiratory therapy, physical therapy, occupational therapy, speech therapy)	5 (0.32)
	Clinical social worker	15 (0.95)
	Dietician or nutritionist	5 (0.32)
	Clinical support (certified medical assistant, emergency medical technician, nurse aide, etc)	12 (0.76)
	Technologist	8 (0.51)
	Technician (eg, surgery, laboratory, radiology)	11 (0.70)
	Admin support (clerk, secretary, receptionist)	23 (1.46)
	Other manager (eg, clinic manager)	260 (16.51)
	Missing	86 (5.46)
**Gender**		
	Male	398 (25.30)
	Female	1100 (69.84)
	Missing	77 (4.89)
**Race and ethnicity**		
	American Indian or Alaska Native	3 (0.19)
	Asian	72 (4.57)
	Black or African American	116 (7.40)
	Hispanic or Latino	88 (5.6)
	Native Hawaiian or other Pacific Islander	4 (0.25)
	White	1165 (73.84)
	Other	40 (2.54)
	Missing	87 (5.52)

### Aim 1: Assessing Change in Well-Being Metrics Between Baseline and 1-Week Follow-Up and Differential Efficacy by Instruction Conditions

Table 2 shows the means and *t* test results across the sample of participants who completed the 1-week follow-up. Examined separately, both conditions showed significant reductions in EE and improvements in happiness and work-life balance (*P*s<.001).

**Table 2:** Independent samples *t* tests comparing changes in well-being for participants in the self- vs other conditions.

Variable	EE^a^ change: self (N=134), mean (SD)	EE change: other (N=135), mean (SD)	*t (df)*	*P* value	95% CI	Happiness change: self (N=136), mean (SD)	Happiness change: other (N=137), mean (SD)	*t (df)*	*P* value	95% CI	Work-life balance change: self (N=132), mean (SD)	Work-life balance change: other (N=134), mean (SD)	*t (df)*	*P* value	95% CI
Self- vs other focus	−7.98 (17.30)	−7.81 (16.85)	−0.08 (257)	.94	−4.27 to 3.93	5.26 (11.01)	4.93 (11.66)	0.24 (471)	.81	−2.37 to 3.04	−0.26 (0.45)	−0.26 (0.42)	−0.12 (264)	.91	−0.11 to 0.10

^a^EE: emotional exhaustion.

**Table 3:** Paired-samples *t* tests assessing well-being across participants who completed the self-focus condition, other-focus condition, and the overall sample at baseline and the 1-week follow-up.

Variable	EE^a^ pre intervention, mean (SD)	EE post intervention, mean (SD)	*t (df)*	*P* value	95% CI	Happiness pre intervention, mean (SD)	Happiness post intervention, mean (SD)	*t (df)*	*P* value	95% CI	Work-life balance pre intervention, mean (SD)	Work-life balance post intervention, mean (SD)	*t (df)*	*P* value	95% CI
Self-focus	61.48 (25.56)^b^	53.51 (27.0)^b^	5.34 (133)	<.001	5.02 to 10.93	67.83 (19.7)^c^	73.09 (18.0)^c^	−5.57 (135)	<.001	−7.12 to −3.39	2.29 (0.61)^d^	2.03 (0.57)^d^	6.90 (131)	<.001	0.19 to 0.34
Other focus	59.88 (26.54)^e^	52.07 (28.48)^e^	5.38 (134)	<.001	4.93 to 10.67	66.84 (19.9)^f^	71.77 (17.4)^f^	−4.95 (136)	<.001	−6.90 to −2.96	2.28 (0.60)^b^	2.02 (0.59)^b^	7.27 (133)	<.001	0.18 to 0.32
Overall 1-week sample	60.67 (26.03)^g^	52.79 (27.7)^g^	7.59 (268)	<.001	5.84 to 9.94	67.34 (19.7)^h^	72.43 (17.7)^h^	−7.43 (272)	<.001	−6.44 to −3.74	2.28 (0.60)^i^	2.02 (0.58)^i^	10.02 (265)	<.001	0.21 to 0.32

^a^EE: emotional exhaustion.

^b^N=134.

^c^N=136.

^d^N=132.

^e^N=135.

^f^N=137.

^g^N=269.

^h^N=273.

^i^N=266.

When directly comparing change in well-being between self- and other-focused conditions, independent *t* tests revealed no significant differences across any of the three well-being measures (*P*s>.05; see Table 2). A linguistic manipulation check revealed that those randomized to the self-focused prompt used more first-person singular words (ie, *I*; self: mean 11.32, SD 4.021; other: mean 10.08, SD 3.66; t_1496_=6.06; *P*<.001) and fewer second-person words (eg, *you*; self: mean 4.61, SD 4.04; other: mean 5.68, SD 4.47; t_1496_=−4.85; *P*<.001) than those randomized to the other-focus condition, indicating that condition prompts did elicit differential linguistic self- vs other focus.

**Figure 1:**
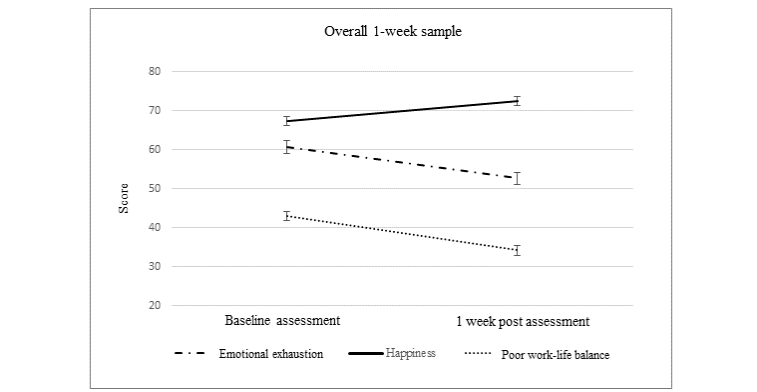
Pre- and postintervention well-being scores. For demonstration purposes, the work-life balance variable was scaled 0-100 in this graph, original response range 1-4. Error bars represent SE means.

As conditions did not differ in their efficacy, we reran the paired *t* tests with the pooled sample to obtain the overall effects, which remained significant (*P*s<.001; see Table 3 and Figure 1). Participants with higher levels of EE at baseline were more likely to be improvers (mean 63.99, SD 23.17) compared with decliners (mean 50.21, SD 26.52) at the 1-week follow-up (t_235_=3.89; *P*<.001). In addition, improvements in specific work-life balance behaviors were examined across the whole sample. Significant improvements were reported across all behaviors (see Table 4), with the biggest improvement in feeling *less* frustration with technology (t_257_=6.18; *P*<.001).

**Table 4:** Paired-samples *t* tests assessing work-life balance items across participants who completed the 1-week follow-up gratitude letter–writing intervention.

Variable	Baseline, mean (SD)	1-week follow-up			
		Mean (SD)	*t (df)*	*P* value	95% CI
Slept less than 5 hours in a night	1.91 (0.94)	1.76 (0.92)	2.68 (260)	.01	0.04 to 0.25
Worked through a shift or day without any breaks	2.32 (1.08)	2.09 (1.04)	4.23 (256)	<.001	0.13 to 0.35
Skipped a meal	2.13 (1.00)	1.88 (0.98)	4.49 (262)	<.001	0.14 to 0.36
Ate a poorly balanced meal	2.63 (0.93)	2.36 (0.99)	5.44 (263)	<.001	0.17 to 0.37
Had difficulty sleeping	2.40 (0.99)	2.10 (1.00)	5.36 (262)	<.001	0.19 to 0.42
Arrived home late from work	2.56 (1.03)	2.24 (0.96)	5.95 (256)	<.001	0.22 to 0.43
Changed personal or family plans because of work	2.07 (0.91)	1.80 (0.83)	5.86 (260)	<.001	0.19 to 0.38
Felt frustrated by technology	2.32 (0.95)	1.97 (0.90)	6.18 (258)	<.001	0.24 to 0.46

### Aim 2: Engagement With the Intervention and Reactions to Getting Emotional Exhaustion Scores

Of 1575 participants who wrote a baseline gratitude letter, 1007 (63.94%) requested an emailed copy of their gratitude letter and 121 (7.68%) reported having completed this gratitude letter exercise before. At the 1-week follow-up, of 140 participants, 136 (56.7%) answered *yes* to the question “Do you have other people in mind for whom you might write a letter like this?” compared with 46 (19.2%) and 58 (24.2%) who answered *no* or *not sure*, respectively. In addition, 75.4% (181/140) participants agreed slightly, strongly, or very strongly with the statement “Since my first gratitude letter a week ago, it has gotten easier to think of things for which I am grateful,” and 45.1% (125/277) participants responded *yes* to the question “Did you talk to anyone about your first gratitude letter?”. Independent *t* tests revealed no significant differences in well-being change scores for those who shared their gratitude letters relative to those who did not (EE: t_266_=1.36, *P*=.18; happiness: t_270_=−1.04, *P*=.30; and work-life balance: t_263_=1.04, *P*=.30).

Participants’ responses to receiving their EE and work-life balance scores can be found in Table 5. It should be noted that 81.23% (1229/1513) of the respondents agreed slightly or strongly with “knowing my burnout score makes me want to work on it more,” and 93.81% (1426/1520) of participants reported wanting to be more resilient.

Those with higher levels of EE at baseline were more likely to agree with all three of these questions (see Table 5). Notably, EE improvers were significantly more likely to agree with the question “Knowing my burnout score makes me want to work on it more” than decliners. Improvers were not more likely than decliners to report being surprised by their scores or a desire to be more resilient.

**Table 5:** Reactions to receiving burnout feedback, differences by baseline emotional exhaustion levels, and emotional exhaustion improvers vs decliners. Responses were on a 1 (disagree strongly) to 5 (agree strongly) scale.

Burnout feedback item	Severe baseline EE^a^ (n=64): those who agreed slightly or strongly, n (%)	Moderate baseline EE (n=515): those who agreed slightly or strongly, n (%)	Mild baseline EE (n=472): those who agreed slightly or strongly, n (%)	No baseline EE (n=473): those who agreed slightly or strongly, n (%)	Concerning EE (n=579) vs no EE (n=473)			EE improvers (n=173) vs decliners (n=62)		
					Mean (SD)	*t (df)*	*P* value	Mean (SD)	*t (df)*	*P* value
“I was surprised by my burnout score”	18 (28.1)	267 (51.8)	211 (44.7)	142 (30)	M1^b^ 3.12 (1.38); M2^c^ 2.72 (1.26)	4.81 (1036.08)	<.001	M1 3.05 (1.25); M2 3.05 (1.26)	−.01 (233)	.99
“Knowing my burnout score makes me want to work on it more”	51 (79.7)	453 (88.5)	400 (85.5)	322 (67.8)	M1 4.50 (0.84); M2 3.92 (1.11)	9.28 (845.30)	<.001	M1 4.39 (0.91); M2 4.03 (1.16)	2.19 (89.10)	.03
“I would like to be more resilient”	62 (96.8)	490 (95.8)	197 (96)	416 (89.1)	M1 4.82 (0.56); M2 4.50 (0.84)	7.07 (761.00)	<.001	M1 4.74 (0.69); M2 4.56 (0.76)	1.64 (231)	.12

^a^EE: emotional exhaustion.

^b^M1: mean numerical response on a 5-point scale for the concerning EE group (moderate and severe EE) at baseline group.

^c^M2: mean numerical response on a 5-point scale for the no EE at baseline group.

### Aim 3: Linguistic Differences Based on Emotional Exhaustion Level at Baseline and Improvements in Emotional Exhaustion

A small number of participants (4.9%) submitted only a few words in the gratitude letter text box. In some cases, it appears that the writer started but did not finish the letter, and in others, the writers engaged in the exercise briefly and superficially (eg, “Mom for raising me. She was amazing.”). As word count scores are computed as a percentage of the total number of words, we were concerned that brief letters could bias the linguistic analyses. To reduce potential bias, we excluded letters in the linguistic analyses that were less than 15 words long (77/1575, 4.89% of the sample).

Linguistic category frequencies were compared for those with concerning levels of EE with those without EE at baseline (see Table 6). Negative emotion words were more frequently used in the gratitude letters by those with concerning levels of EE at baseline (*P*=.005). All other word categories were not used differentially by baseline EE level (*P*>.05).

**Table 6:** Independent *t* tests of linguistic differences in gratitude letters by baseline emotional exhaustion (concerning or not) and emotional exhaustion improved (or declined) at the 1-week follow-up.

Hypothesized categories	Baseline EE^a^				EE improvement 1 week later			
	EE concerning, mean (SD)	EE not concerning, mean (SD)	*t (df)*	*P* value	EE decliners (n=63), mean (SD)	EE improvers (n=175), mean (SD)	*t (df)*	*P* value
First person (eg, *I*, *I’ve*, and *my*)	10.96 (4.52)	10.91 (5.10)	0.17 (1070)	.87	11.88 (4.59)	10.65 (4.24)	−1.93 (236)	.06
First-person plural (eg, *we*, *let’s*, and *us*)	0.74 (1.48)	0.84 (1.69)	−1.01 (981.69)	.31	0.82 (1.46)	0.81 (1.65)	−0.03 (236)	.98
Negative emotion (eg, *annoy*, *angry*, and *scream*)	1.16 (1.54)	0.92 (1.36)	2.76 (1066.53)	.005	1.04 (1.56)	1.02 (1.42)	−0.10 (236)	.92
Positive emotion (eg, *appreciate*, *funny*, and *thank*)	8.61 (4.71)	8.61 (5.20)	−0.032 (1070)	.98	9.88 (7.27)	8.51 (4.56)	−1.73 (236)	.09
Cognitive processing (eg, *accept*, *because*, and *realization*)	12.13 (5.28)	11.89 (5.42)	0.73 (1070)	.47	11.52 (5.28)	12.06 (5.27)	0.70 (236)	.48

^a^EE: emotional exhaustion.

The frequencies of word categories were compared for EE improvers vs decliners. There was a statistical trend, albeit nonsignificant, showing that improvers used fewer first-person and positive emotion words (*P*=.06 and .09, respectively). Otherwise, EE improvers vs decliners did not demonstrate different use across the remaining word categories.

## Discussion

### Principal Findings

This study examined the efficacy of a single-use gratitude letter–writing intervention with a diverse sample of health care workers. Between baseline and the 1-week follow-up, participants reported significant improvements in all three of the well-being measures: EE, happiness, and work-life balance. Participants across both randomized instruction conditions (self-focus vs other focus) reported equivalent improvements in well-being. To our knowledge, this is the first application of a onetime gratitude intervention in health care workers evidencing subsequent improvement in the EE component of burnout.

Participants reported high engagement with the intervention among those who completed the follow-up. Three-fourths of the participants (75.4%) reported that it was easier to think of things to be grateful for since writing their letter. A majority (63.9%) of participants requested an emailed copy of their letter, presumably to reflect on it and/or share with the recipient. Participants equally benefited from the tool regardless of whether they spoke with someone about the letter or not. Although nearly half (45.1%) of the participants reported talking to someone about their letter, we do not know if that person was the letter recipient or not. Previous research has indicated that writing a letter should be followed by a visit to the recipient of the letter [16,17]. It may be that speaking with the letter recipient confers additional benefits, whereas speaking with someone in general (recipient or not) about their letter does not.

Our intervention also gave participants immediate feedback about their EE score during the baseline assessment. Participants were split between being surprised and not being surprised by their scores; however, participants who had higher EE scores were more likely to be surprised. This suggests that a significant portion of health care workers may be burned out, yet they are either not aware of it or are in denial about it [36,37]. After receiving their scores, participants reported very high levels of motivation to reduce their EE (81.2%) and increase their resilience (93.8%). Receiving one’s EE score might trigger behavioral change to improve well-being. Indeed, higher agreement to the question “Knowing my burnout score makes me want to work on it more” predicted greater improvement in EE. We cannot determine whether this improvement was because of behavioral change that occurred between the assessments or whether individuals who had higher agreement to this question experienced greater benefit from the gratitude letter–writing exercise or because of some other reason. However, considerable research has shown that greater awareness of a personal problem is a key step in behavioral change [38,39]. A recent meta-analysis of burnout prevalence in 109,628 physicians across 182 studies suggests that many physicians are answering burnout questions, but we believe that the proportion receiving feedback about their burnout is close to 0 [40]. It could be that burnout psychoeducation on its own may not be sufficiently motivating. Rather, receiving one’s burnout score can be likened to someone *stepping on the scale* before engaging in more physical activity, bringing a personal awareness of his or her status to trigger behavioral change.

Linguistic analyses revealed that participants with moderate and severe EE at baseline used more negative emotion words in their letters compared with participants without EE. Similarly, previous research has shown that depressives use more negative emotion words in their writing samples [19]. Surprisingly, we did not find differences in the use of first-person singular or plural, positive emotions, or cognitive processing word categories by EE severity. Although we found a statistical trend associating greater reductions in EE with the use of fewer first-person singular words (eg, *I* and me) and fewer positive emotion words, neither of them reached statistical significance (*P*=.06 and *P*=.09, respectively; see the Multimedia Appendix 1). Differences in these categories have been found in depressives vs controls in prior research, albeit with very different writing prompts [19]. Writing a note of gratitude might draw out a different pattern of language compared with the expressive writing prompts often used by other linguistics researchers, which ask participants to write about their deepest emotions and emotional challenges over a 4-day period [41]. Thus, linguistic patterns in the gratitude letter tool may not reflect psychological well-being as clearly as was found in other studies. Given that the gratitude tool appears to be beneficial, it may be that an overall feeling of gratitude induced by this intervention drives improvement, rather than a complex linguistic pattern across word categories. In other words, it may simply be more important *that* you are grateful rather than *how* you are grateful.

In line with prior research, we expected that participants who were assigned to write more about themselves, rather than the recipient of the letter, would benefit more from the exercise. Although our prompts appeared to change the linguistic focus (ie, the self-focused condition used more *I* and less *me* language than the other-focused condition), one condition did not further enhance well-being relative to the other. Notably, prior research on the benefits of self-focused language examined verbal expressions of gratitude, rather than written letters [18]. It may be that in a letter format, the impact of focusing on oneself during expressions of gratitude is inherently weaker or diluted by the additional time spent writing.

This study and prior research indicate that the gratitude letter tool, and similar positive psychology tools, might improve well-being by shifting one’s focus from what is *not* going well to what *is* going well. Indeed, the negativity bias makes people significantly more likely to pay attention to negative vs positive stimuli [42]. Individuals with higher levels of negativity bias are more likely to be depressed [43]. When prompted to positively reflect, participants appear to consider good things they had previously taken for granted, had not thought much about, or were formerly overshadowed by attention to negative experiences. By intentionally shifting attention to what is going well, participants may recalibrate their perceptions toward noticing more positive experiences and be primed to engage in other self-care activities such as exercising [44]. Indeed, 75.4% of follow-up participants reported that it became easier to think of things to be grateful for after writing their first gratitude letter.

We chose to implement gratitude letter writing as a web-based intervention to increase the availability of the tool and increase our sample size. In fact, our baseline sample size was larger than we anticipated. However, it is possible that a web-based format made it easier for participants to ignore the 1-week follow-up survey message. Web-based studies typically have high dropout rates, likely because of the ease of ignoring the request and reduction of interpersonal reciprocity norms. It is possible that personal characteristics make web-based tools more appealing to some, and this could have influenced enrollment and engagement in the study. Future research should explore what characteristics predispose individuals to benefit from web-based vs in-person well-being tools.

### Limitations

Our study has several limitations. First, our study included randomization to two letter conditions, but there was no nonintervention control group. In addition, the intervention included both the gratitude letter activity and receiving one’s baseline EE score, component effects of which we cannot disentangle. These aspects preclude our establishing a causal relationship between gratitude letter writing and improvement in well-being. However, prior randomized controlled gratitude interventions (eg, nightly gratitude log and gratitude visit) have demonstrated improvement compared with placebo controls, which bolsters confidence in the results of this study [16].

Nonetheless, the observed benefits of this intervention may in part arise from expectancy bias or placebo effects. We observe that participants who engaged in the gratitude letter tool and completed the 1-week follow-up reported significant improvements across all outcomes. It is possible that there were intrinsic characteristics of the cohort that completed follow-up assessments that predisposed them to improvement, whereas participants not completing postintervention assessments may not have benefited to the same degree.

Selection bias comprises another noted limitation. Our sample largely comprised women (69.9% of participants at baseline). Nevertheless, this approximates the base rates of women working in health care in the United States (in 2017, 75% of health care workers were female) [45]. Future research should employ a placebo-controlled design, examine the gratitude letter or EE feedback alone, and potentially oversample for men, all of which will serve to better determine causality, divine specific effects of the tool, and establish the extent of efficacy across genders. Overall, the high level of engagement observed within our cohort, significant participant interest in discussing letters, and self-reported improvements in the ability to identify that for which they were grateful suggest a meaningful impact of this short letter-writing activity.

Our study also suffered from what looks like attrition, with 18% of participants who completed a baseline assessment also completing the 1-week follow-up. A significant component likely arose from varied means by which health care workers were referred to the tool. In some cases, participants received a brief link at the conclusion of a talk or meeting, without mention of any follow-up evaluation: “Consider giving this just 7 minutes to see what you think of a bite-sized resilience activity.” In other words, although many participants enrolled knowing that there would be a 1-week follow-up, some participants likely never intended on completing the follow-up at the outset. It is possible that individuals who failed to complete the follow-up did not benefit from the tool to the same extent as those who did complete it, overinflating the tool’s effects. That said, our attrition rates are similar to comparable studies [46-48]. Attrition is a primary barrier to evaluating web-based interventions, with levels often reaching 60% to 80%. Among people seeking treatment for obesity using weight loss programs in medical centers, one-third to half discontinue their program and are lost to follow-up [49,50]. Similarly, over 40% of people seeking treatment through a smoking cessation clinic were lost to follow-up [51], and 48% of web-based smoking cessation individuals were lost to follow-up [52]. Even when offered significant financial incentives (US $600) to participate in a cessation program, only 20% of those invited chose to participate [53]. In future iterations of this tool, we plan to improve the follow-up rates by setting clear expectations for receiving and completing follow-up assessments, identifying whether participants correctly entered their contact information into our system, and implementing reminders.

Perhaps most importantly, the gratitude letter tool is designed to build participants’ personal well-being resources but does not address the demands placed upon them in their work environments. Ultimately, the pace and intensity of contemporary health care are major unaddressed sources of strain that impair well-being. The gratitude letter tool, although promising, should be part of a much larger toolkit of valid and accessible interventions facilitating individual well-being and institutional changes.

Despite these limitations, this study finds that the gratitude letter tool offers a simple, brief, and free individual intervention to improve health care workers’ EE, happiness, and work-life balance. The flexibility of this single-use tool is highly desirable amid the fast pace of health care. Indeed, it can be inserted into a staff meeting, during a break, or completed briefly at home. In an era of alarmingly high levels of health care worker EE, the gratitude letter tool appears to be a promising arrow in the quiver to improve well-being.

### Conclusions

A single-exposure gratitude letter–writing intervention is a promising tool to improve EE, happiness, and work-life balance in health care workers. There is preliminary evidence that receiving feedback about your EE serves to motivate further well-being action and, in turn, improve EE. The gratitude letter tool [23] is brief, simple, and free and requires no further action after writing a 7-min letter, which are all important factors for busy health care workers and overburdened health care systems. Given the relationship between health care workers’ well-being and health care quality, engaging in the gratitude letter tool may help relieve EE and, in turn, improve patient safety and quality.

## Appendix

Multimedia Appendix 1 Discussion on trending linguistics results.

## Abbreviations

EE: emotional exhaustion
LIWC: Linguistic Inquiry and Word Count
MBI: Maslach Burnout Inventory
SHS: Subjective Happiness Scale
