# Supplementary material for: Gratitude at Work: Prospective Cohort Study of a Web-Based, Single-Exposure Well-Being Intervention for Health Care Workers
Source: J Med Internet Res. 2020 May 14;22(5):e15562. doi: 10.2196/15562 (PMC7256751; doi:10.2196/15562)
Supplement: Multimedia Appendix 1 [file jmir_v22i5e15562_app1.docx]

Online Supplemental for:

Gratitude at Work: A Prospective Cohort Study of a Brief, Single-exposure Well-being

Intervention for Healthcare Workers

Discussion on Trending Linguistic Results

Participants reporting greater reductions in EE trended toward using fewer first person singular words (e.g., “I”, “me”) in their letters (p = .055). It’s thought that greater physical or emotional pain draws writers’ attention to themselves. Indeed, suffering with depression and suicidality^40^ predicts greater first-person singular use in other writing samples.^29^ Our findings suggest that those who have less self-focus, reflected by less first person singular use at baseline, might be more likely to benefit from the gratitude letter tool.

Contrary to hypothesis, EE Improvers used slightly fewer positive emotion words in their letters, again however at the level of a trend (p = .085). We expected positive emotion expression to facilitate greater reductions in burnout, given the salutary effect that experiencing positive emotions can have for mental health.^41^ Perhaps participants who used more positive emotion words wrote more relatively superficial letters compared to those who were focusing on more poignant or meaningful aspects of gratitude. Future coding of gratitude letters may shed light on the role of positive emotions within the tool. We report and interpret both of these findings for EE Improvers with caution, however, since they did not meet thresholds of statistical significance.
